# Affinity- and Specificity-Enhancing Mutations Are Frequent in Multispecific Interactions between TIMP2 and MMPs

**DOI:** 10.1371/journal.pone.0093712

**Published:** 2014-04-07

**Authors:** Oz Sharabi, Jason Shirian, Moran Grossman, Mario Lebendiker, Irit Sagi, Julia Shifman

**Affiliations:** 1 Department of Biological Chemistry, The Alexander Silberman Institute of Life Sciences, The Hebrew University of Jerusalem, Jerusalem, Israel; 2 Department of Biological Regulation, Weizmann Institute of Science, Rehovot, Israel; 3 Wolfson Center for Structural Biology, The Alexander Silberman Institute of Life Sciences, The Hebrew University of Jerusalem, Jerusalem, Israel; German Research School for Simulation Science, Germany

## Abstract

Multispecific proteins play a major role in controlling various functions such as signaling, regulation of transcription/translation, and immune response. Hence, a thorough understanding of the atomic-level principles governing multispecific interactions is important not only for the advancement of basic science but also for applied research such as drug design. Here, we study evolution of an exemplary multispecific protein, a Tissue Inhibitor of Matrix Metalloproteinases 2 (TIMP2) that binds with comparable affinities to more than twenty-six members of the Matrix Metalloproteinase (MMP) and the related ADAMs families. We postulate that due to its multispecific nature, TIMP2 is not optimized to bind to any individual MMP type, but rather embodies a compromise required for interactions with all MMPs. To explore this hypothesis, we perform computational saturation mutagenesis of the TIMP2 binding interface and predict changes in free energy of binding to eight MMP targets. Computational results reveal the non-optimality of the TIMP2 binding interface for all studied proteins, identifying many affinity-enhancing mutations at multiple positions. Several TIMP2 point mutants predicted to enhance binding affinity and/or binding specificity towards MMP14 were selected for experimental verification. Experimental results show high abundance of affinity-enhancing mutations in TIMP2, with some point mutations producing more than ten-fold improvement in affinity to MMP14. Our computational and experimental results collaboratively demonstrate that the TIMP2 sequence lies far from the fitness maximum when interacting with its target enzymes. This non-optimality of the binding interface and high potential for improvement might characterize all proteins evolved for binding to multiple targets.

## Introduction

Virtually all functions in the cell are regulated through cascades of protein-protein interactions (PPIs). Some biological processes cause activation of several parallel PPI pathways that frequently intertwine with each other. At the crossroads of such pathways lie proteins that are capable of interacting with a number of different partners and hence are called multispecific proteins [1]. Due to their central role in PPI networks, multispecific proteins are crucial to cell survival and their malfunction inevitably leads to disease. Thus, unraveling the atomic-based principles for binding multispecificity is not only interesting for basic biology but also valuable for the studies directed at finding new therapeutics that target various PPIs. Binding interface sequences of multispecific proteins are under evolutionary pressure to provide favorable interactions for various partners that in some cases share little sequence and structure homology. These sequences are a compromise required for accommodating multiple targets and thus cannot be optimal for interactions with each individual target protein. We postulate that binding interface sequences of multispecific proteins lie far from the fitness maximum for each individual interaction and thus could be further improved through mutations. In other words, mutations that enhance binding affinity should be frequent in multispecific PPIs. Moreover, such mutations are likely to narrow down binding specificity of multispecific proteins towards a particular target or a set of targets.

**Figure 1:**
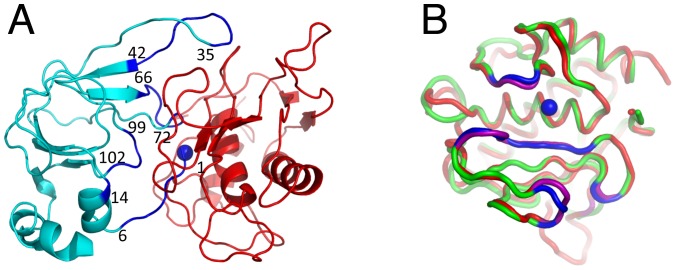
Structural Analysis of MMP/N-TIMP interactions. (A) MMP-14 interacting with N-TIMP2 (PDB ID 1BUV). MMP14 is shown in red, N-TIMP2 – in cyan. The catalytic Zn^2+^ ion is shown as a blue sphere. The interacting regions on N-TIMP2 are colored in blue and their boundaries are numbered. (B) N-TIMP2 binding interface on MMPs. Superposition of backbones for MMP14 (red) and MMP9 (green). The regions that contact N-TIMP2 are shown in purple for MMP14 and in blue for MMP9. MMP14 and MMP9 exhibit 59% sequence identity and 70% sequence similarity in the binding interface region and exhibit Cα RMSD of 0.66 Å. [39]

To test this hypothesis, we chose a representative multispecific protein, Tissue Inhibitor of Metalloproteinases 2 (TIMP2). TIMP2 is one of four similar proteins in humans (TIMP1, 2, 3 and 4) that regulate a family of more than twenty-six homologous enzymes, Matrix Metalloproteinases (MMPs) and the related ADAMs families [2]–[4]. MMPs play a major role in degradation of the extracellular matrix and participate in many important biological processes such as embryonic development, organ morphogenesis, bone remodeling and others. On the other hand, imbalance in MMP activity is associated with a diverse set of diseases including arthritis, cardiovascular diseases, neurological disorders, fibrosis, and cancer [5]. MMPs are multi-domain proteins that differ in domain architecture and substrate preferences [6] but all share a catalytic domain with a nearly identical active site containing a Zn^2+^ ion. High-resolution structures have been solved for a number of MMPs alone and in complex with TIMPs [7]–[13].[Murphy, 2011 #643] These structures reveal that TIMPs bind directly to the catalytic zinc ion at the active site of the enzyme, shielding it from the solvent. The interaction is conveyed mostly through the TIMP N-terminal domain (N-TIMP) consisting of ∼125 residues. N-TIMP is a potent inhibitor of various MMPs and thus has been repeatedly used as a substitute for the full-length protein in various experimental studies [14]. N-TIMP binds to MMPs mostly through four contiguous regions (Figure 1A). The first region includes six N-terminal residues that come in close proximity to the enzyme active site and coordinate the catalytic Zn^2+^ through the N-terminal Cys. Besides the N-terminal region, three additional N-TIMP loops (35–42, 66–72, and 97–99 in N-TIMP2) participate in direct interactions with MMPs (Figure 1A).

MMPs are synthesized in an inactive form. They could be activated by other MMPs and inactivated upon binding of TIMPs. Each of the four known TIMPs binds most of the MMPs with slightly different affinities, ranging from 10^−11^–10^−9^ M. In addition, some TIMPs, such as TIMP-2, can participate in the activation path of certain MMPs, through binding to another MMP type [15]. TIMP/MMP interactions hence present a complicated regulatory network with connections that are not fully understood. Rational manipulation of this network through mutations could help to elucidate precise functional roles of various MMPs and facilitate development of selective inhibitors for each MMP type. TIMPs present an attractive scaffold for design of such inhibitors and hence have been a subject of various mutational studies. Previous studies demonstrated that certain substitutions at positions 2, 4, and 68 of TIMP2 can strongly affect its relative affinity for different MMPs [16]–[18]. In another study, a single mutation T98L that stabilizes TIMP1 in the bound conformation was shown to produce an impressive specificity shift towards MMP-14 relative to other MMP types [19], [20]. More recently, phage display technology was used to probe a large number of possible mutations in the N-TIMP2 binding interface and to engineer a variant that binds to MMP1 with a nanomolar affinity while losing its affinity to MMP3 and MMP14 [21].

In contrast to previous studies, our goal was to obtain a more comprehensive picture of TIMP/MMP interactions and to locate positions on TIMP where affinity- and specificity-enhancing mutations could be identified with high probability. For this purpose, we first generated computational binding landscapes of N-TIMP2/MMP14 and N-TIMP2/MMP9 interactions by predicting effects of all single mutations in the N-TIMP2 binding interface on its affinity to these two enzymes. We validated some of our predictions experimentally by constructing a number of N-TIMP2 mutants and measuring their affinity to these two enzymes. We extended our computational studies to six additional MMPs for which structural models for interactions with TIMP2 could be constructed. Both computational and experimental results point to the suboptimal nature of the N-TIMP2 binding interface sequence and possibility of affinity and specificity improvement through various mutations. These results are in agreement with our hypothesis that multispecific proteins are not optimized for a particular binding partner and could be reengineered to be more selective interaction partners and inhibitors.

## Materials and Methods

### Model construction for N-TIMP2/MMP complexes

We created models for structures of MMP/TIMP2 complexes for those MMPs that have their X-ray structure available only in the unbound form (MMP1 (PBD 3SHI), MMP2 (PDB 1RTG), MMP3 (PDB 1B3D), MMP7 (PDB 1MMQ), and MMP9 (PDB 1L6J)). For this purpose, we first superimposed the unbound structure of a particular MMP on the structure of the MMP14/N-TIMP2 complex (PDB 1BUV). We next removed from the structure all MMP residues that do not belong to the catalytic domain. This initial superimposed structure of the MMP/N-TIMP2 complex was then refined using the RosettaDock server [22]. The best output structure from the RosettaDock server was used as an input for the saturation mutagenesis protocol.

### Computational saturation mutagenesis

An *In silico* saturation mutagenesis protocol was applied on the N-TIMP2 binding interface using the structure of the N-TIMP2/MMP-14 (PDB code 1BUV) complex [8], the N-TIMP2/MMP13 (PDB code 2E2D) complex [9], and the N-TIMP2/MMP10 complex (PDB code 4ILW) and the models constructed for the N-TIMP2/MMP complex. Only the N-terminal TIMP2 domain was used in the calculations (residues 1–127 of TIMP2). The metal ions Ca^2+^ and Zn^2+^ were not considered in the calculations. The N-TIMP2 binding interface was defined as all the residues that are within 4 Å from the MMP in the N-TIMP2/MMP14 structure and included residues 1–4, 6, 14, 35, 36, 38, 40, 42, 66, 68, 69, 70, 71, 97, 99, and 100–101. From this set we excluded positions that are very close to the catalytic zinc ion (positions 1–3, and 100–101) and positions that coordinate a calcium ion (position 36) since effects on catalysis and interactions with ions could not be adequately modeled by our protocol. The remaining fourteen residues were scanned using the saturation mutagenesis protocol described in Sharabi et al [23]. Briefly, for each of the fourteen positions, we performed 18 calculations where the considered position on N-TIMP2 was either kept WT or was replaced with another amino acid, all except for Pro, Cys, and Gly. During the calculation, the interface residues as well as residues in direct contact with the interface were repacked and the energy of the N-TIMP2/MMP complex was calculated for the WT and for the mutated complex. We then separated the two chains and calculated the energy of each single chain. The intermolecular energy was calculated by subtracting the energies of the single chains (N-TIMP2 and MMP) from the total energy of the complex. ΔΔG_bind_ was calculated by subtracting the intermolecular energy of the WT complex from the intermolecular energy of the mutant. Finally, the obtained ΔΔG_bind_ was normalized according to a linear equation obtained in our previous work where correlation between various experimental and computed ΔΔG_bind_ values was tested [24]. Rotamer libraries used for design were based on the backbone dependent library of Dunbrack and Karplus [25] with additional rotamers expanded by one standard deviation around their mean χ_1_ and χ_2_ values. For the calculations, we used ORBIT software with the energy function optimized by our group for design of protein-protein interactions [24]. The energy function contained terms that describe Van der Waals attractive and repulsive interactions, hydrogen bond interactions, electrostatic interactions, and surface-area-based solvation (see [24] for the exact description of the energy function,). The lowest-energy rotameric conformation of each mutant was found using the Dead-End Elimination theorem [26], [27]. Finally, we color-coded each mutation according to its ΔΔG_bind_ value in kcal/mol: ΔΔG_bind_ ≥1.5 - red; 0.5≤ΔΔG_bind_ <1.5 – yellow; −0.5≤ΔΔG_bind_ <0.5 - green and ΔΔG_bind_ ≤−0.5 - blue. Mutations that were predicted to destabilize unbound N-TIMP2 or an unbound MMP by more than 2 kcal/mol were considered potentially deleterious for N-TIMP2 folding and were colored in gray if predicted to improve ΔΔG_bind_.

### Evaluating N-TIMP2 position tolerance and specificity potential

We evaluated tolerance of each N-TIMP2 binding interface position for mutations based on the results of the saturation mutagenesis protocol for ΔΔG_bind_ prediction. For this purpose, we replaced each color saturated mutagenesis figure a score: −1 for blue, 0 for green, 1 for yellow, and 2 for red mutations. Gray mutations were not incorporated in the calculation. We calculated the average score over all mutations at a single binding interface position for one MMP and assigned positions into three classes according to the score: Score ≤0.2 → tolerant, 0.2<Score≤1 → semi-tolerant, Score >1 → non-tolerant. To evaluate the potential of a particular mutation to narrow down binding specificity, we compared ΔΔG_bind_ predictions for one particular mutation among the eight MMP types. For each particular mutation, we calculated the average score and its standard deviation over all MMPs. A mutation with standard deviation greater than 1 was considered beneficial for enhancing binding specificity over all eight MMPs and was marked by a star. In addition, we calculated an average score and standard deviation over all mutations for each N-TIMP2 position.

### MMP enzymes

Catalytic domains of MMP14 and MMP9 were expressed recombinantly and purified as published before [12].

### Expression and refolding of the N-TIMP2 mutants

Genes for the N-TIMP-2 mutants were generated by the Transfer PCR protocol [28] starting from the plasmid pET-28a-*timp-2-HISX6* containing the gene for the WT N-TIMP2 (residues 1–127). TIMP2 mutants were expressed in *E. coli* BL21 (DE3) cells (Novagen) as described previously [14]. N-TIMP2 variants were extracted from inclusion bodies by sonication with 50 mM Tris-HCl, pH 8.75 and 6 M Gnd-HCl and incubated with 10 mM DTT for 1.5 hours. The solution was slowly dripped into 1 mM/0.5 mM reduced/oxidized glutathione, 0.5 M Gnd-HCl and 50 mM Tris-HCl, pH 8.75, to a final concentration of 100 μg/ml. The sample was left at 4°C overnight. On the following day, the sample was loaded on a Ni-column, washed three times with a buffer containing 50 mM Tris-HCl, 0.3 M NaCl, 10 mM Imidazole, pH 7.5. The protein was eluted with a buffer containing 50 mM Tris-HCl, 0.3 M NaCl, 250 mM Imidazole, pH 7.5. The monomeric fraction of N-TIMP2 was separated using gel filtration with the Superdex 75 10/300 GL column equilibrated with 50 mM Tris, 100 mM NaCl, 5 mM CaCl_2_, pH 7.5.

### Binding affinity determinations using the enzyme activity essay

The synthetic fluorogenic MMP substrate MCA-Pro-Leu-Gly-Leu-Dpa-Ala-Arg-NH_2_ ·TFA [where MCA is (7-methoxycoumarin-4-yl)acetyl; Dpa is N-3-(2,4-dinitrophenyl)-L-2,3-diaminopropionyl; and TFA is trifluoroacetic acid] was purchased from GenScript Inc., (Piscataway, NJ) and used to assay enzyme activity. Samples with various concentrations of the N-TIMP2 mutant were pre-incubated with either MMP9 (at 0.2 nM concentration) or with MMP14 (at 0.5 nM concentration) for 1 hour in the buffer containing 50 mM Tris-HCl, 0.15 M NaCl, 10 mM CaCl2, and 0.02% Brij 35 pH 7.5 at 37°C. A 50 μl aliquot of substrate (15 μM) was added to 150 μl of the pre-incubated MMP/N-TIMP2 mixture and the enzyme activity was measured on a TECAN infinite m-200 microplate reader by exciting at 325 nm and measuring fluorescence at 395 nm. The reaction was measured for the initial 20 minutes where product release was linear with time. Fraction of enzyme activity *f* was calculated by dividing the slope of the reaction in the presence of the N-TIMP2 inhibitor by the slope of the reaction in the absence of N-TIMP2 inhibitor. K_d_ values were then fitted from the data assuming a 1∶1 binding model according to the equation:
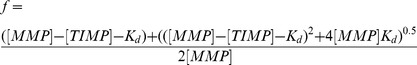
(1)


Where [MMP] and [TIMP] are concentrations of MMP and N-TIMP2, respectively.

## Results

### Mapping computational binding landscapes for N-TIMP2/MMP14 and N-TIMP2/MMP9 interactions

Each protein-protein interaction can be characterized by a binding landscape that represents changes in protein-protein binding affinity due to point mutations. To generate the binding landscape of the N-TIMP2/MMP14 and N-TIMP2/MMP9 interactions, we used the computational saturation mutagenesis protocol developed in our lab [29]. This protocol scans each PPI binding interface position with all amino acids, repacks the surrounding side chains and determines the change in free energy of binding due to mutations (ΔΔG_bind_) (see Methods). As an input for the protocol, we used an X-ray structure of the N-TIMP2/MMP14 complex and a structural model of the N-TIMP2/MMP9 complex generated from the structure of unbound MMP9 (see Methods). While usage of a structural model instead of an actual structure is bound to introduce some inaccuracies in our calculations, we were optimistic in the case of MMP9 since this enzyme exhibits high structural homology to MMP14 in the N-TIMP2 binding region with a Cα RMSD of 0.66 Å for interfacial residues (Figure 1B). Next, we computationally scanned fourteen N-TIMP2 positions with seventeen amino acids, producing binding energy landscapes for the N-TIMP2/MMP14 and the N-TIMP2/MMP9 interactions. We did not consider mutations to Gly and Pro since those mutations are likely to result in backbone conformational changes that were not modeled by our protocol. Mutations to Cys were not considered due to their possible interference with the correct disulphide bond formation in N-TIMP2.

**Figure 2:**
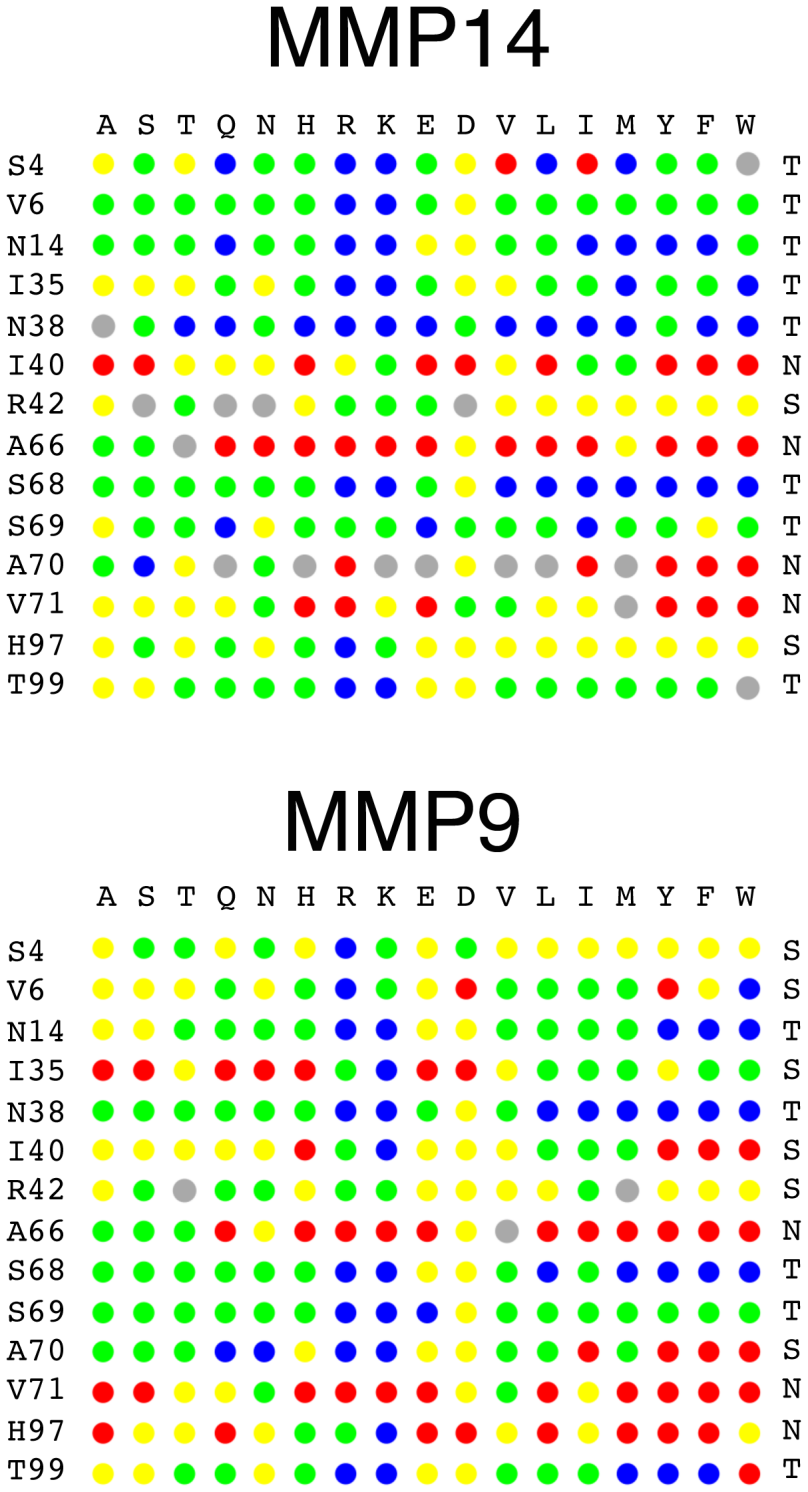
Computational binding landscapes for the N-TIMP2/MMP14 (A) and the N-TIMP2/MMP9 (B) interactions. N-TIMP2 binding interface positions with their WT identity are displayed on the left, the mutated amino acid identity is on the top. Calculated ΔΔG_bind_ value for each mutation is color coded: ΔΔG_bind_ ≥1.5 kcal/mol: red, 0.5 kcal/mol ≤ ΔΔG_bind_ <1.5 kcal/mol: yellow, 0.5 kcal/mol ≤ ΔΔG_bind_ < −0.5 kcal/mol: green and ΔΔG_bind_ ≤ −0.5 kcal/mol: blue. Mutations where negative ΔΔG_bind_ is coupled to significant destabilization of a single chain (>2 kcal/mol) are shown in gray. For these mutations we cannot reliably predict ΔΔG_bind_. Positions are divided into tolerant, semi-tolerant and non-tolerant denoted by T, S, and N on the right of the figure.

To better visualize the binding energy landscape of the N-TIMP2/MMP14 complex, each mutation was assigned to one of four classes according to the predicted ΔΔG_bind_ value and colored in blue, green, yellow, and red for affinity-enhancing, neutral, destabilizing, and highly destabilizing mutations, respectively (Figure 2). In addition, we classified each N-TIMP2 binding interface position according to its ability to accept various mutations into tolerant, semi-tolerant and non-tolerant (see Methods).

Figure 2 shows that the N-TIMP2 interface is not particularly optimized for binding to either MMP since the landscape is dominated by neutral and affinity-enhancing mutations represented by green and blue circles. For example, for MMP-14, out of fourteen considered N-TIMP2 binding interface positions, ten showed possibility of significant ΔΔG_bind_ improvement with mutation to at least one amino acid (Blue circles, Figure 2). For six such positions, significant ΔΔG_bind_ improvement was predicted for mutations to three or more different amino acids. Moreover, eight positions were predicted as tolerant, two positions were predicted as semi-tolerant and the remaining four positions were predicted as non-tolerant. Although effects of particular mutations on N-TIMP2 are different between MMP14 and MMP9, the N-TIMP2/MMP9 binding landscape is qualitatively similar to that of the N-TIMP2/MMP14 interactions, showing many possibilities for affinity improvements, five tolerant positions, six semi-tolerant positions, and three non-tolerant positions.

### Experimental testing of computational predictions

**Figure 3:**
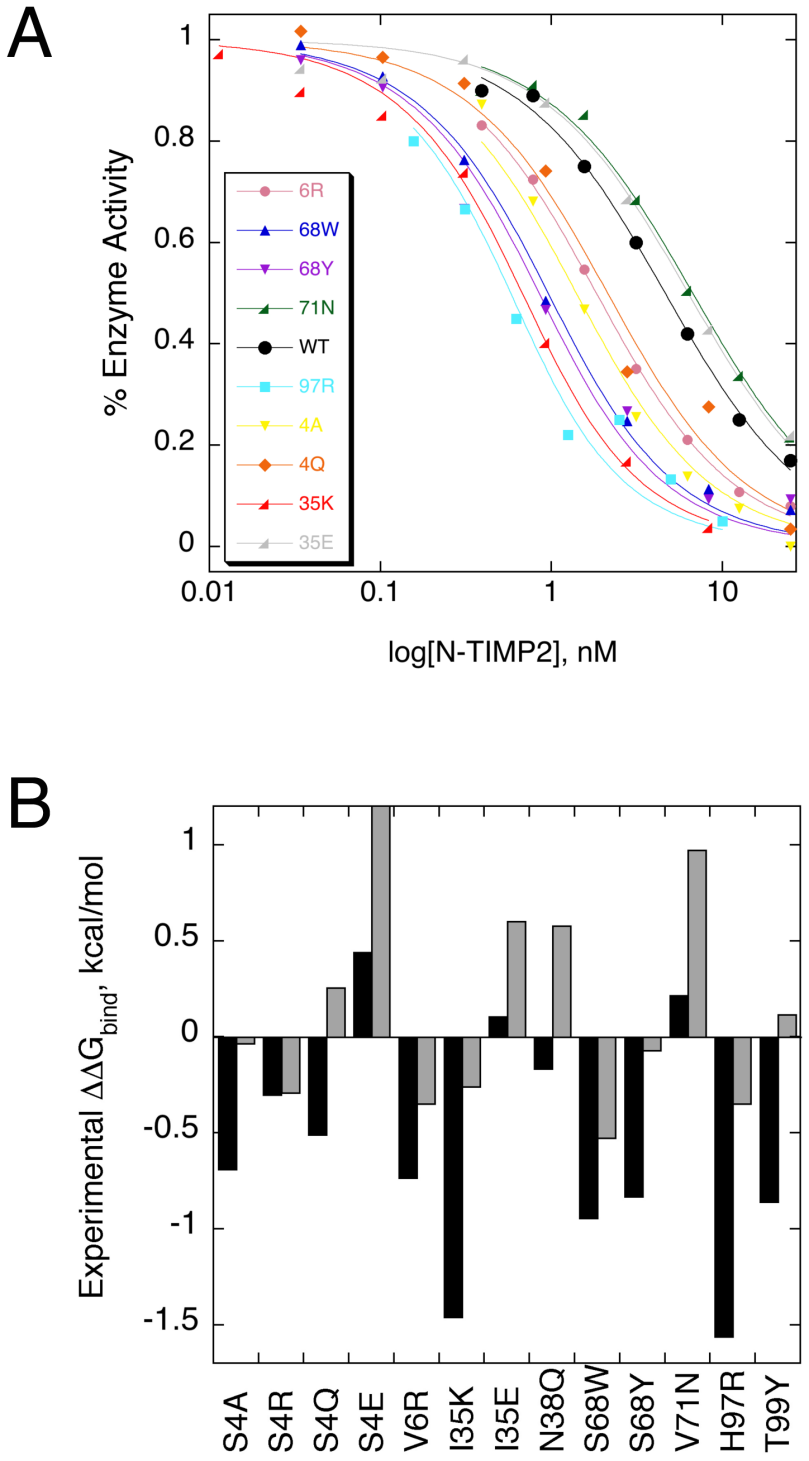
Binding affinity measurements between N-TIMP2 mutants and MMP14/MMP9. (A) Enzyme activity assay is performed in the presence and the absence of N-TIMP2 and the fraction of enzyme activity is plotted vs. log of N-TIMP2 concentration. The curves were fitted to equation 1 to determine K_d_ of the interaction. (B) ΔΔG_bind_ calculated from the K_d_ measured in (A) for each studied N-TIMP2 mutation when interacting with MMP14 (black bars) and with MMP9 (gray bars).

To determine how well our computational binding landscapes reflect the reality of the TIMP2/MMP binding energetics, we decided to validate some of the predictions experimentally. The number of the tested N-TIMP2 mutants was limited by a relatively tedious procedure for their construction that requires refolding after expression in *E. Coli* [14]. We hence selected thirteen N-TIMP2 single mutants, focusing on mutations that 1) were predicted to enhance binding affinity to MMP14 and 2) were predicted to enhance binding specificity towards MMP14 relative to MMP9 (Table 1). To measure binding between the N-TIMP2 mutants and MMP14/MMP9, we utilized an enzyme activity assay described previously (Figure 3A) [30]. This assay is based on detecting fluorescence resulting from cleavage of a fluorogenic MMP substrate. High sensitivity of the assay allows us to measure binding affinities as low as 10^−11^ M.

**Table 1:** Predicted and measured changes in binding affinity.

N-TIMP2 mutant	K_d_ for MMP14, nM^1^	K_d_ for MMP9, nM^1^	Experimental ΔΔG_bind_ to MMP14, kcal/mol	Experimental ΔΔG_bind_ to MMP9, kcal/mol	Computational ΔΔG_bind_ MMP14, kcal/mol	Computational ΔΔG_bind_ MMP9, kcal/mol	Measured specificity shift^2^	Specificity shift is predicted correctly^3^
WT	4.5±0.2	0.9±0.2	N. A.	N. A.	0	0	N. A.	N.A.
S4A	1.4±0.3	0.85±0.22	−0.69	−0.03	0.50	1.45	3.0	+
S4R	2.7±0.1	0.55±0.05	−0.31	−0.29	−3.30	−0.54	1.1	−
S4Q	1.90±0.02	1.39±0.14	−0.51	0.26	−1.60	0.93	3.7	+
S4E	9.5±1.0	15.5±1.2	0.44	1.68	−0.25	1.37	8.6	+
V6R	1.30±0.19	0.50±0.12	−0.73	−0.35	−2.13	−0.53	1.9	+
I35K	0.38±0.13	0.58±0.08	−1.46	−0.26	−1.83	−1.07	7.1	+
I35E	5.4±0.6	2.50±0.13	0.11	0.60	0.09	1.73	2.4	+
N38Q	3.4±±0.6	2.40±0.55	−0.17	0.58	−1.19	0.10	3.3	+
S68W	0.91±0.18	0.37±0.04	−0.94	−0.52	−2.21	−0.70	2.0	+
S68Y	1.1±0.18	0.8±0.2	−0.83	−0.07	−1.23	−0.78	3.6	+
V71N	6.5±0.4	4.68±0.08	0.22	0.97	−0.38	0.20	3.6	+
H97R	0.32±0.05	0.50±0.03	−1.56	−0.35	−1.48	−0.39	7.7	+
T99Y	1.05±0.11	1.1±0.3	−0.86	0.12	0.24	−1.21	4.5	−

1 All experiments were repeated 3 times and average and standard deviation for K_d_ is reported

2 The specificity shift was defined as the fold of affinity enhancement towards MMP14, divided by the fold of affinity enhancement towards MMP9.

3 The specificity is considered predicted correctly if the calculated difference between ΔΔG_bind_ for the N-TIMP2 mutant/MMP14 complex and the N-TIMP2 mutant/MMP9 complex agrees in sign with the experimental difference between the respective ΔΔG_bind_ values.

Using the above assay, we determined K_d_s for interactions between N-TIMP2 WT and MMP14 and MMP9 to be 4.5 and 0.9 nM respectively, similar to previously published results [21]. These K_d_s became a point of reference for calculating ΔΔG_bind_ for the selected N-TIMP2 mutants. Eight mutations that were predicted to significantly improve binding affinity of N-TIMP2 to MMP14 (S4R, S4Q, V6R, I35K, N38Q, S68W/Y, H97R) proved to be affinity-enhancing experimentally (Table 1 and Figure 3B). In addition, two mutations, S4A and T99Y that were predicted as neutral or slightly destabilizing also showed improved binding affinity for MMP14. Among the affinity-enhancing mutations two, I35K and H97R, exhibited a 12- and 14-fold improvement in binding affinity towards MMP14, an impressive affinity shift for single mutations. Five of the N-TIMP2 mutants with increased affinity towards MMP14 (S4R, V6R, I35K, S68W, and H97R) also exhibited affinity enhancement towards MMP9 (Figure 3B and Table 1). This demonstrates that affinity-enhancing mutations at the N-TIMP2 binding interface could be easily found through our computational protocol. Twelve out of thirteen explored N-TIMP2 mutants produced a detectible shift in binding specificity towards MMP14 relative to MMP9 (Table 1). Three of the mutations produced a substantial (7–9 fold) shift in binding specificity towards MMP14 relative to MMP9, including mutations I35K and H97R that also improved affinity towards this enzyme and S4E that was slightly destabilizing for the complex with MMP14 and highly destabilizing for the complex with MMP9 (Table 1). We hence conclude that both affinity- and specificity-enhancing mutations are quite frequent at the N-TIMP2 binding interface. Good consensus of our predictions with experimental results for MMP9, where no actual X-ray structure of the complex was available, gave us confidence that fairly realistic binding landscapes could be constructed with our computational saturation mutagenesis protocol using structural models of PPIs as starting points. We next tested whether our findings on low-optimality of the N-TIMP2 binding interface could be extended to additional MMP types.

### Computational binding landscapes of N-TIMP2 interacting with additional MMPs

**Figure 4:**
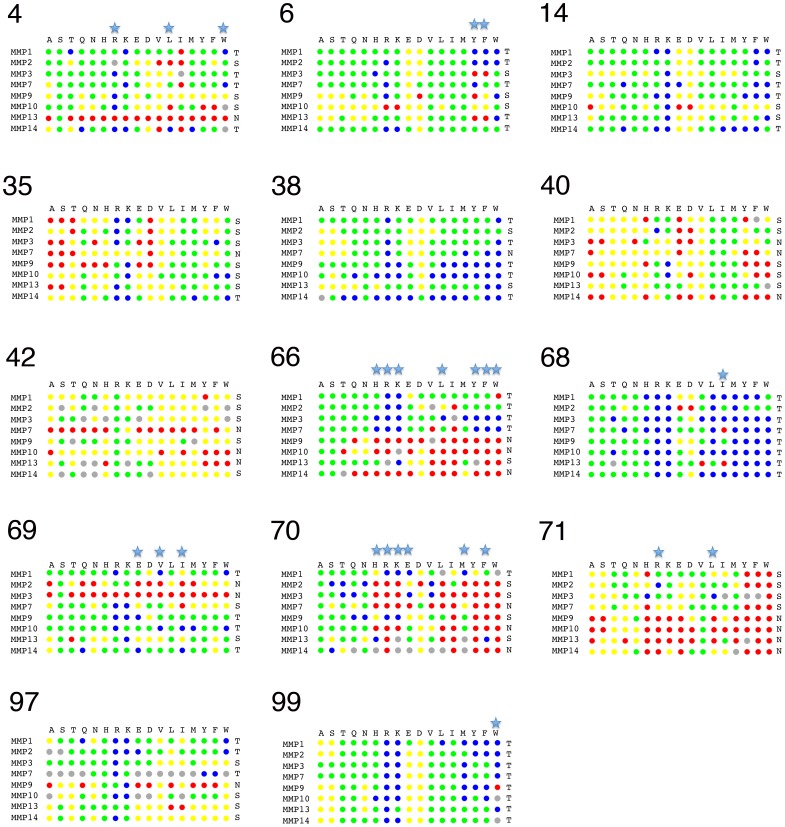
Per-position ΔΔG_bind_ predictions for N-TIMP2 interacting with eight studied MMPs. Color coding is the same as in Figure 2. Mutations with the standard deviation greater than one are marked by stars (see Methods for calculation of the standard deviation).

We aimed to explore N-TIMP2 computational binding landscapes for as many MMPs as possible. The limiting factor here was the structural information on the N-TIMP2/MMP complexes. Presently, three high-resolution structures of the TIMP2/MMP complexes are available (for MMP10, MMP13 and MMP14). Nevertheless, we were able to generate additional structural models of the N-TIMP2 complexes for those MMPs that have their structure solved in the unbound form including: MMP1, MMP2, MMP3, and MMP7. Together with MMP14 and MMP9, we thus analyzed computational binding landscapes for eight MMP family members. Figure 4 compares the effect of mutations at each of the N-TIMP2 positions for interactions with eight MMPs. Figure 4 shows that most of the positions on N-TIMP2 can accommodate a large number of mutations while preserving or even improving binding to various MMPs. The less optimized positions include 14, 68, and 99, where affinity-enhancing mutations were found for all studied MMPs. Less tolerant positions include 35, 40, 42, 70, and 71 where the majority of substitutions lead to decrease in affinity. However, even at these positions a few choices of neutral and affinity-enhancing mutations were observed. When averaging the results over eight MMPs, the N-TIMP2 binding interface contains 6.125 tolerant positions, 5.125 semi-tolerant positions, and 2.75 non-tolerant positions, revealing suboptimal nature of interactions for all studied N-TIMP2/MMP complexes. Most of the tolerant positions, such as, for example position 68, lie on the periphery of the binding interface and show only partial contacts with MMPs. The non-tolerant positions, on the other hand, such as positions 70 and 71, lie in the core of the binding interface where various MMP residues pack tightly against them.

## Discussion

### Low optimality of the N-TIMP2/MMP binding landscapes

Our computational and experimental findings point to the relatively low optimality of the N-TIMP2/MMP interfaces. The *in silico* saturation mutagenesis protocol predicted that affinity enhancement could be produced through at least three different mutations at eight different N-TIMP2 positions when interacting with MMP14 and at six different positions when interacting with MMP9 (Figure 2). Experimentally, with only a few trials, we identified affinity-enhancing mutations at seven and five positions for MMP14 and MMP9, respectively (Table 1). Computational binding landscapes of the remaining MMPs also point to high potential for affinity improvement (Figure 4). The low optimality of the N-TIMP2/MMP interfaces is not surprising since N-TIMP2 binds to all MMP members with similar affinities and hence cannot provide favorable intermolecular interactions for each MMP. Our results are in agreement with previous computational studies of multispecific proteins whose binding interface sequence was found to be optimal for simultaneous interactions with different targets yet sub-optimal for interaction with each target on its own [31], . An ability to greatly improve binding affinity and specificity through only a few mutations was recently experimentally demonstrated in ubiquitin, a protein whose function is to bind to multiple targets with low affinity [33]. In contrast, our recent study on high-affinity enzyme-inhibitor complexes revealed highly optimized binding landscapes with only a handful of mutations that further increase affinity [34]. All of the above findings suggest that low optimality of the binding interface might be a general property of multispecific interactions that distinguishes them from PPIs with narrow binding specificity.

### Analysis of affinity-enhancing mutations

**Figure 5:**
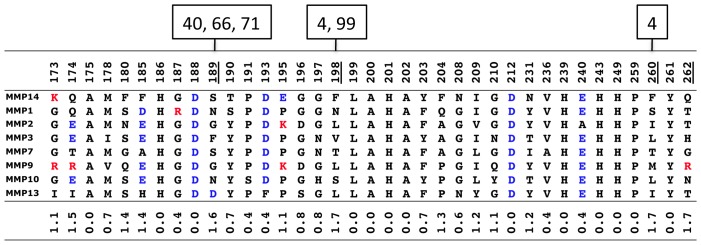
Structure-based sequence alignment of the N-TIMP2 contacting residues for eight MMPs under study. Negatively charged amino acids are colored blue while positively charged residues are colored red. Shannon entropy [39] that represents sequence variability at a particular position is shown below. N-TIMP2 positions with the entropy greater or equal to 1.6 are underlined. Positions on N-TIMP2 that contact these high-entropy positions (among those explored in this work) are shown on top of the table above the corresponding MMP position.

**Figure 6:**
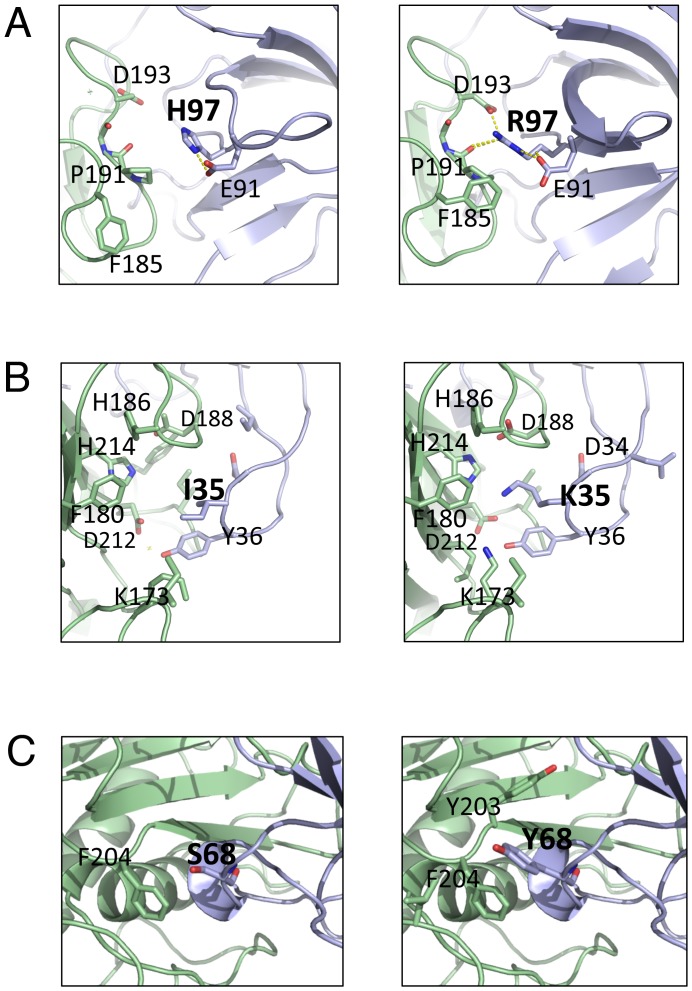
Structural analysis of the affinity-enhancing mutations. H97R (A); I35K (B); S68Y (C). The left panel shows WT interaction and the right panel shows interaction after mutation. N-TIMP2 is shown in blue and MMP14 is shown in green. Mutated residues and surrounding residues are shown as sticks and hydrogen bonds and salt bridges are shown as yellow dots.

Eight out of ten experimentally identified affinity-enhancing mutations were correctly predicted for the N-TIMP2/MMP14 interaction and four out of five mutations were correctly predicted for the N-TIMP2/MMP9 interaction, demonstrating the potential of our *in silico* saturation mutagenesis approach in identifying affinity-enhancing mutation and its applicability not only to crystal structures but also to structural models. Among the identified mutations two, I35K and H97R, exhibited affinity improvement of more than ten-fold, which is higher than usually observed for single mutations [35]. Both of our best affinity-enhancing mutations are substitutions to positively charged residues. This is not surprising since the MMP binding interface is slightly negatively charged (Figure 5). In addition, both substitutions occur at positions, where no significant interaction with MMP14 occurs in the wild-type N-TIMP2/MMP14 complex while favorable intermolecular interactions are created upon substitution. Substitution of His to Arg at position 97 forms additional Van der Waals interactions and creates new intermolecular hydrogen bonds and electrostatic interactions with Asp 193 on MMP14 and with the backbone carbonyl (Figure 6A). Mutation of an Ile at position 35 to Lys also improves interface packing and creates favorable electrostatic interactions with Asp 188 and Asp 212 on MMP14 (Figure 6B). Other identified affinity-enhancing mutations (S68W, S68Y and T99Y) are mutations from small to aromatic amino acids that fill in the gaps in the non-optimal interface and bury additional hydrophobic area (Figure 6C). Burial of larger hydrophobic area has been proposed as a strategy for selecting affinity-enhancing mutations in a previous study [36].

### Analysis of specificity-enhancing mutations

All but one tested N-TIMP2 mutants exhibited a shift in binding specificity towards MMP14 relative to MMP9, in agreement with most of our predictions (Table 1). While the observed specificity shift was modest, combining such mutations might result in an N-TIMP2 mutant that shows many-fold preference for one MMP type. To better understand how binding specificity is conveyed at the molecular level, we analyzed all mutations in the structural context, by looking at modeled complexes between N-TIMP2 mutants and MMP14/MMP9. Our analysis showed that most such mutations created specific interactions [37], [38], such as hydrogen bonds, salt bridges, or pi-pi stacking interactions with MMP14, but were unable to form similar interactions with MMP9 due to the absence of an appropriate amino acid on the enzyme side (Table 2). For example, Tyr 99 is predicted to form a pi-pi stacking interaction with F198 on MMP14, but lacks an aromatic interaction partner on MMP9. Similarly, Gln 38 is predicted to form a hydrogen bond with Q208 on MMP14; this residue is replaced by a Gly on MMP9, thus disallowing any hydrogen bond interaction.

**Table 2:** Structural analysis of interactions of various N-TIMP2 mutants with MMP14 and MMP9.

N-TIMP2 mutation	S04E	S04Q	S04R	V06R	I35K	N38Q	S68W/Y	V71N	H97R	H99Y
Predicted HBs^1^/SBs^2^ with MMP14	N231	N231	E195 E195, E219	E195 E195	H214 D212, D188	Q208		H201 Y203	D193 D193	
Predicted pi-pi interactions with MMP14 residues							Y203, F204			F198
Respective AA on MMP9	Y393	Y393	K184	K184		G197	F192, P193	H190, F192		L187

1 HBs, hydrogen bonds between N-TIMP2 mutant and MMP14 side chains

2 SBs, salt bridges between N-TIMP2 mutant and MMP14 side chains.

Based on the computational binding landscapes of N-TIMP2/MMP interactions generated in this work, we further propose a strategy for selecting specificity-determining positions, or positions where mutations have a high potential for narrowing down binding specificity. Such positions, (e. g. positions 4, 35, 66, 69, 70, and 71) display high standard deviation in ΔΔG_bind_ predictions across the whole MMP family (Table S1). Interestingly, half of these positions (4, 66, 71) are also in contact with positions on MMP that exhibit the highest sequence variability over eight MMP types (Figure 5). These specificity-determining positions should be the focus of experiments that rely on selection of N-TIMP mutants with narrowed specificity from large combinatorial libraries of mutants.

Furthermore, using computational binding landscapes we can predict specific mutations that narrow down N-TIMP2 binding specificity for certain MMP types (Figure 4, indicated by stars). For example, mutation V6Y is predicted to significantly destabilize N-TIMP2 interactions with MMP3, MMP9, MMP13 while at the same time stabilizing its interactions with MMP1, MMP2, MMP7. Mutation V71R on the other hand is predicted to improve interactions with MMP2, while destabilizing complexes with MMP7, MMP9, MMP13, and MMP14. Note that predictions of specific mutations are more sensitive to inaccuracies in our computational protocol compared to predictions at the position and the interface level. The latter predictions reflect the global picture and depend only slightly on the results for each individual mutation.

In summary, we demonstrated that the N-TIMP2 binding interface is not optimal for binding to various MMPs, revealing a large number of mutations that improve binding affinity towards a particular MMP type. This sub-optimality might be a general property of mutispecific PPIs that have evolved to provide reasonable affinity for a large set of targets. It is thus relatively easy to enhance binding affinity of a multispecific protein towards one particular target, and the affinity increase is frequently coupled to an increase in binding specificity.

## Supporting Information

Table S1 **Position Specificity Potential.** Average score and standard deviation over all mutations at a single binding interface position for all MMPs is presented.
(DOCX)
