# Supplementary material for: Affinity- and Specificity-Enhancing Mutations Are Frequent in Multispecific Interactions between TIMP2 and MMPs
Source: PLoS One. 2014 Apr 7;9(4):e93712. doi: 10.1371/journal.pone.0093712 (PMC3977929; doi:10.1371/journal.pone.0093712)
Supplement: Table S1 — Position Specificity Potential. Average score and standard deviation over all mutations at a single binding interface position for all MMPs is presented. (DOCX) [file pone.0093712.s001.docx]

| Supplementary Table S1  Position Specificity potential | |
| --- | --- |
| N-TIMP2 position | Standard deviation^1^ |
| 70 | 1.124278 |
| 66 | 1.101048 |
| 69 | 0.938617 |
| 4 | 0.916886 |
| 35 | 0.889554 |
| 71 | 0.863642 |
| 97 | 0.822156 |
| 40 | 0.781019 |
| 68 | 0.753447 |
| 14 | 0.728201 |
| 6 | 0.719838 |
| 99 | 0.712208 |
| 38 | 0.689088 |
| 42 | 0.654168 |

^1^Standard deviation for each position was calculated by first replacing each color on Figure 4 by an appropriate score: -1 for blue, 0 for green, 1 for yellow, and 2 for red mutations. Gray mutations were not incorporated in the calculation. Average score and standard deviation over all mutations at a single binding interface position for all MMPs was calculated.
